# Effectors of Root-Knot Nematodes: An Arsenal for Successful Parasitism

**DOI:** 10.3389/fpls.2021.800030

**Published:** 2021-12-22

**Authors:** Shounak Jagdale, Uma Rao, Ashok P. Giri

**Affiliations:** ^1^Plant Molecular Biology Unit, Division of Biochemical Sciences, CSIR-National Chemical Laboratory, Pune, India; ^2^Academy of Scientific and Innovative Research (AcSIR), Ghaziabad, India; ^3^Division of Nematology, ICAR-Indian Agricultural Research Institute, New Delhi, India

**Keywords:** root-knot nematode, effectors, oesophageal glands, giant cells, plant-nematode interaction

## Abstract

Root-knot nematodes (RKNs) are notorious plant-parasitic nematodes first recorded in 1855 in cucumber plants. They are microscopic, obligate endoparasites that cause severe losses in agriculture and horticulture. They evade plant immunity, hijack the plant cell cycle, and metabolism to modify healthy cells into giant cells (GCs) – RKN feeding sites. RKNs secrete various effector molecules which suppress the plant defence and tamper with plant cellular and molecular biology. These effectors originate mainly from sub-ventral and dorsal oesophageal glands. Recently, a few non-oesophageal gland secreted effectors have been discovered. Effectors are essential for the entry of RKNs in plants, subsequently formation and maintenance of the GCs during the parasitism. In the past two decades, advanced genomic and post-genomic techniques identified many effectors, out of which only a few are well characterized. In this review, we provide molecular and functional details of RKN effectors secreted during parasitism. We list the known effectors and pinpoint their molecular functions. Moreover, we attempt to provide a comprehensive insight into RKN effectors concerning their implications on overall plant and nematode biology. Since effectors are the primary and prime molecular weapons of RKNs to invade the plant, it is imperative to understand their intriguing and complex functions to design counter-strategies against RKN infection.

## Introduction

**Figure 1:**
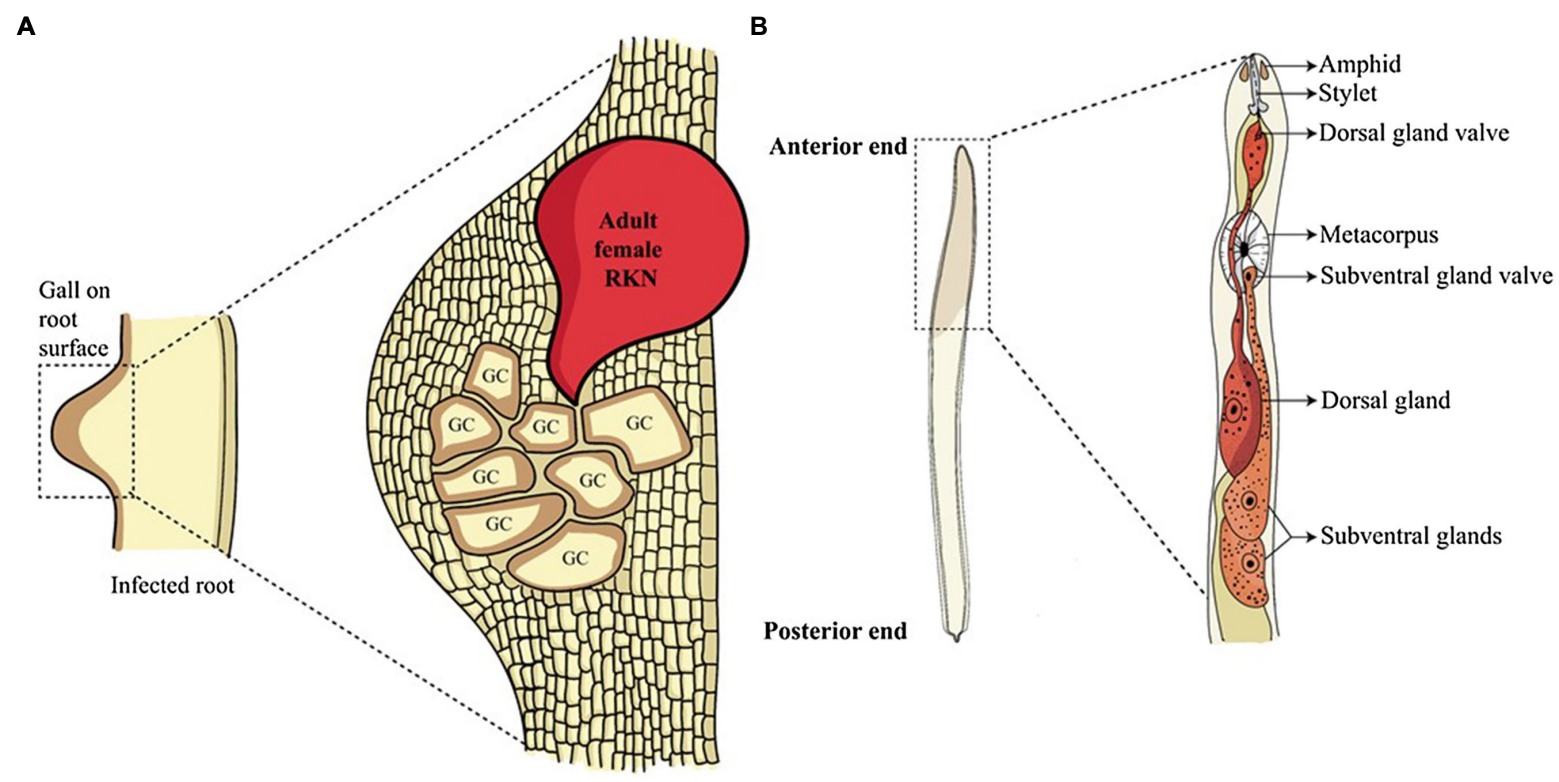
Root-knot nematode infection and glands involved in effector synthesis. **(A)** Root-knot nematode infected roots show a gall-like structure on the root surface. Inside the gall, adult sedentary nematodes reside. These adults secrete a large number of effectors in the plant cells converting them into multinucleated GCs, the feeding sites of nematodes. **(B)** The anatomy of the anterior structure of pre-parasitic juvenile RKN shows two main oesophageal glands. The oesophageal glands, namely sub-ventral glands (SvGs) and dorsal gland (DG) are the primary sites of effector production in nematodes. The effectors produced by these glands are secreted through the stylet into the plant cells.

Root-knot nematodes (RKNs) are ubiquitous, obligate, biotrophic plant-endoparasites of the genus *Meloidogyne* spread across tropical and subtropical regions. About 100 species of *Meloidogyne* are known to attack more than 3,000 plants species causing multibillion-dollar annual losses (Gowda et al., 2017; Forghani and Hajihassani, 2020). Their life cycle varies between 3 to 6 weeks based on species and environmental conditions. Adult females lay about 500 to 1,000 eggs in a gelatinous matrix which is secreted on the root surface. Under favourable conditions, second-stage juveniles (pre-parasitic J2s) hatch out of eggs which is the only infective stage that enter the plant roots near the root-tip. Once inside, they (parasitic J2s) migrate through intercellular spaces until they reach the root meristem. Here parasitic J2s make a U-turn and migrate up the vascular cylinder and become sedentary on reaching the protoxylem. At this point, they select 5 to 8 cells and pump them with various effectors secreted from the oesophageal glands resulting in the cellular reprogramming and the formation of giant cells (GCs; Figure 1A; Abad and Williamson, 2010).

**Figure 2:**
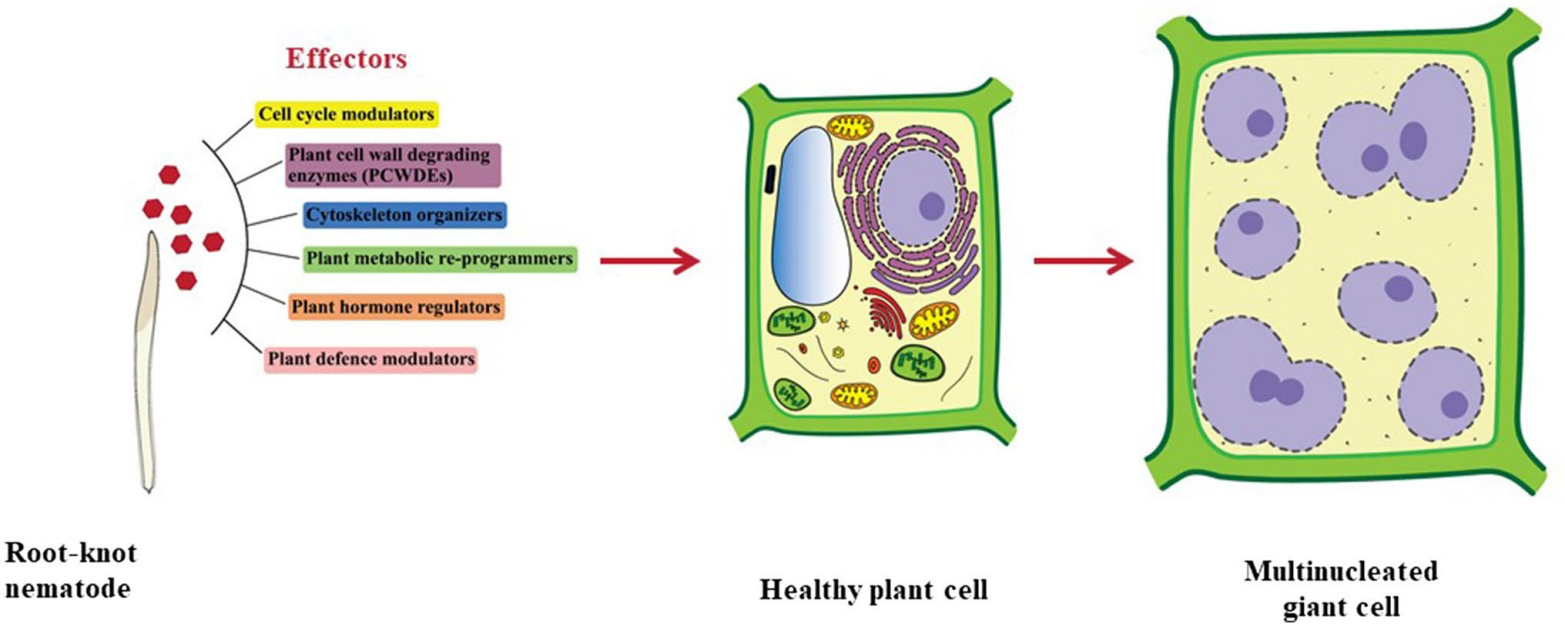
Types of effectors secreted by RKNs. Effectors secreted by RKNs are divided into various categories namely cell cycle modulators, PCWDEs, cytoskeleton organizers, plant metabolic re-programmers, plant hormone regulators, and plant defence modulators. Various effectors from these categories work in tight coordination to modify normal plant cells into GCs.

To enter the plant root and develop the GCs, RKNs use two vital weapons. First is the brute force of their stylet to mechanically and enzymatically break the plant cell wall; second is the effectors secreted through the oesophageal glands to manipulate host cell metabolism. RKNs have two types of oesophageal glands: a pair of the sub-ventral glands (SvGs) and a dorsal gland (DG; Figure 1B). Temporally, SvGs are active during the early stage of infection (pre-parasitic and parasitic J2s) and DG becomes functional during early and late infection (parasitic J2s to adults). Effectors are important molecules deployed by pests and pathogens to facilitate the infection and nutrient acquisition (Carreón-Anguiano et al., 2020). Described RKN effectors are mostly proteinaceous molecules secreted to target host molecular components to enable parasitism (Vieira and Gleason, 2019). With the help of recent developments in pan-omics and bioinformatics tools, many effectors have been identified (Jaubert et al., 2002b; Huang et al., 2003, 2004; Abad et al., 2008; Bellafiore et al., 2008; Opperman et al., 2008; Rehman et al., 2016; Shukla et al., 2018; Vieira and Gleason, 2019). Initially, *M. incognita* was the most studied RKN for effectors. However, recently many experimentations on *M. javanica*, *M. graminicola*, *M. arenaria*, *M. enterolobii* and *M. chitwoodi* have increased the numbers of known effectors. The effectors secreted by RKNs are classified into various groups based on their function during infection *viz*.: plant cell wall degrading enzymes (PCWDEs), plant defence modulators, plant hormone regulators, cell cycle modulators, cytoskeleton organizers, and plant metabolic re-programmers (Figure 2; Favery et al., 2020). However, many effectors have no homology to known proteins making the RKN infection process complex to elucidate. Various tedious and complex experimental methodologies such as yeast 2-hybrid, pull-down experiments, RNAi approach, and *in-situ* strategies have been utilized to implicate their molecular functions (Eves-van den Akker et al., 2021). In the past decade, few non-oesophageal effectors have also been identified increasing the layers of complexity of plant-RKN interactions (Danchin et al., 2013; Rutter et al., 2014; Zhao et al., 2019).

During the past 5 years, over 50 reviews have discussed the general biology, ecology, and evolution of plant-parasitic nematodes (Kikuchi et al., 2017; Siddique and Grundler, 2018; Kaloshian and Teixeira, 2019; Hewezi, 2020; Eves-van den Akker, 2021). Of these reviews, eight articles describe the effectors of plant-parasitic nematodes (Ali et al., 2017; Mejias et al., 2019; Perrine-Walker, 2019; Sato et al., 2019; Vieira and Gleason, 2019; Favery et al., 2020; Eves-van den Akker, 2021; Eves-van den Akker et al., 2021). These reviews summarize the effector-mediated molecular and physiological changes in plants (Ali et al., 2017; Mejias et al., 2019; Perrine-Walker, 2019; Favery et al., 2020; Eves-van den Akker, 2021), plant immune responses against nematodes (Sato et al., 2019), and new effector discovery tools (Vieira and Gleason, 2019; Eves-van den Akker et al., 2021). However, to the best of our knowledge, none of the reviews focuses exclusively on RKN effectors. Hence, in this review, we have compiled the recent advances in the understanding of the molecular and biochemical mechanisms of exclusively RKN effectors during the process of parasitism so as to summarise the extent of progress and identify the gaps for cracking the Pandora box of nematode infection process. First, we discuss the effector discovery strategies, then provide the molecular information of various RKN effectors, their site of biosynthesis along with localization in plant organelles and expression kinetics during nematode infection process and development. We begin with effectors from SvGs and how they allow pre-parasitic and parasitic J2s to initiate the infection process. This is followed by the role of DG effectors secreted by the sedentary stages of RKNs. We also highlight the functions of effectors and their effects on overall plant immunity, metabolism, and cell cycle. Finally, we discuss the non-oesophageal effectors and their roles in parasitism. Thus, we provide a comprehensive insight into the RKN infection process, where the specific role of effectors is described at the molecular level.

## Strategies and Success in the Discovery of Effectors From Rkns

The obligate sedentary mode of parasitism along with microscopic nature of RKNs are significant impediments to study their secretory proteins. Different strategies like differential gene expression, cDNA library screening, and direct analysis of secretory proteins employed in the late 1990s to study the RKN effectors were of limited success (Bird, 2004). In 2003, direct micro-aspiration of cytoplasm from *M. incognita* oesophageal glands and cDNA sequencing identified 37 effectors (Huang et al., 2003). Later solid-phase subtractive hybridization was used to identify and clone the effector genes from *M. incognita* (Huang et al., 2004). The first genomic approach of EST sequencing of pre-parasitic *M. incognita* J2s revealed several cell wall degrading enzymes (McCarter et al., 2003). In 2008, the first draft genome of *M. incognita* identified various putative effectors (Abad et al., 2008). In subsequent years sequencing of other RKN species *viz., M. hapla*, *M. javanica*, *M. arenaria*, *M. floridensis*, *M. graminicola*, and *M. enterolobii* boosted the effector discovery rate (Opperman et al., 2008; Blanc-Mathieu et al., 2017; Szitenberg et al., 2017; Sato et al., 2018; Somvanshi et al., 2018, 2021; Koutsovoulos et al., 2020). Genome-wide searches of proteins with signal peptides was done to identify putative effectors. Furthermore, genome-wide transcriptomic analyses of pre-parasitic J2s helped to identify putative effector genes that were upregulated specifically during early parasitic stages (Dubreuil et al., 2007; Haegeman et al., 2013). Later, dual RNAseq allowed the discovery of effectors in a stage-specific manner (Li et al., 2016; Petitot et al., 2016, 2020; Shukla et al., 2018; Grynberg et al., 2020). Recently, life stage specific transcriptomics combined with available genome data gave insights into spatio-temporal regulation of *M. incognita* effector expression (Da Rocha et al., 2021). In addition to these, *in situ* hybridization in specific regions of the nematode body provided significant impetus for validating the effectors (Roze et al., 2008; Haegeman et al., 2013; Rutter et al., 2014; Gleason et al., 2017; Nguyen et al., 2018). Presently, *M.incognita* stands as the most studied RKN species with more than 100 putative oesophageal effector genes being reported (Da Rocha et al., 2021). The details about the molecular functions of the well/partially characterized effectors from *M. incognita* and other RKNs are described in the following sections.

## Role of Sub-Ventral Oesophageal Glands in Early Infection

**Figure 3:**
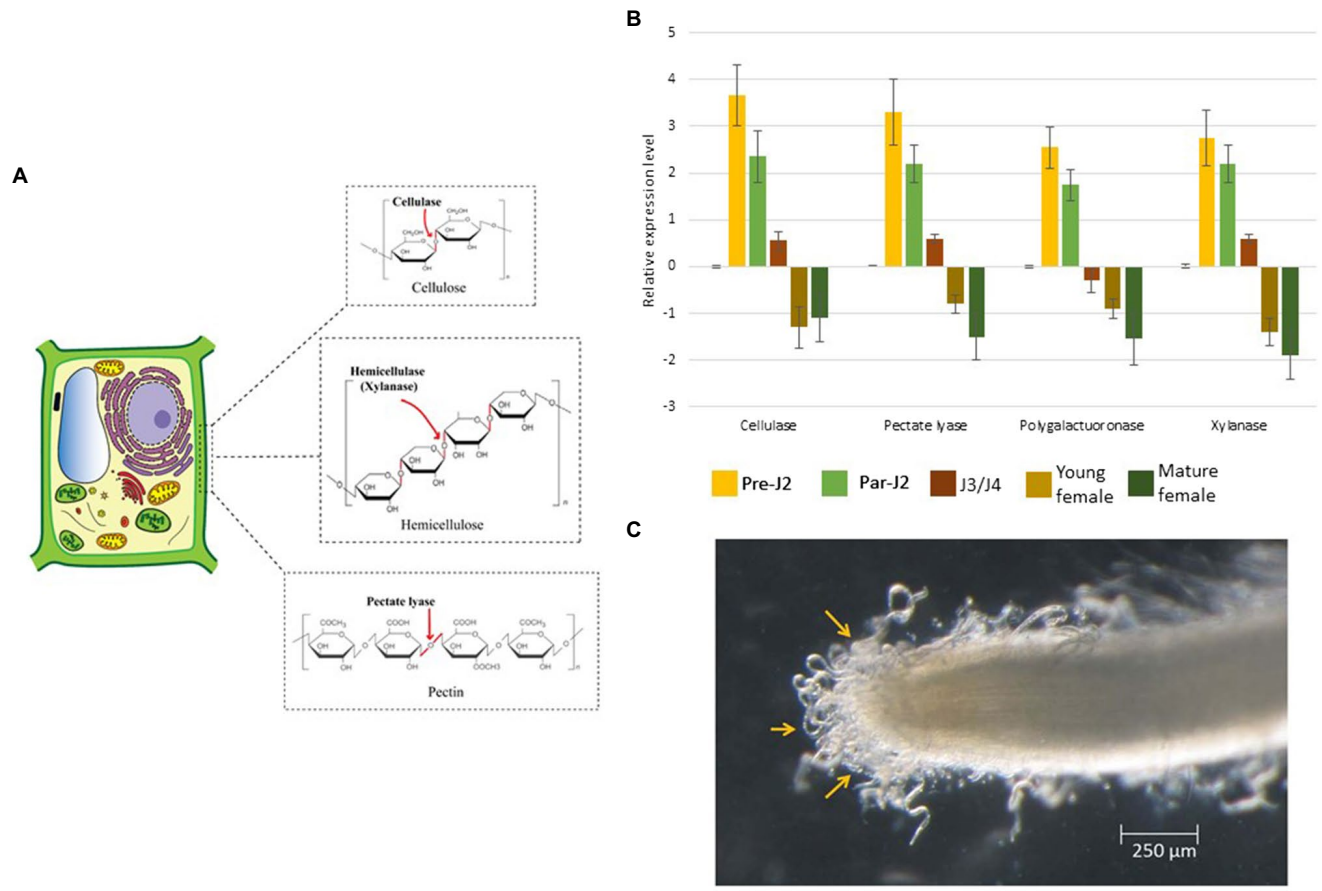
Molecular effects of CWDEs. **(A)** Pre-parasitic juveniles produce a cocktail of CWDEs in the SvGs. Cellulases act on cellulose, the major backbone of the plant cell wall. Xylanase cleaves hemicellulose. Pectate lyase acts on pectin which weakens the cell wall. **(B)** The real-time expression profile of CWDEs shows maximum expression during pre-parasitic J2s which reduces with RKN development. The expression data is derived from (Shivakumara et al., 2017). About 400 ng RNA was used to prepare cDNA. The transcript level in all the stages was compared with that in eggs. Expression level was quantified using 2^−ΔΔCt^ method. 18S rRNA was used as a reference gene. **(C)** Pre-parasitic juveniles latched to the root surface prior to entering the root. The RNAseq analysis of pre-parasitic J2s at this stage in different host plants could reveal the CWDE isoform expression dynamics. The image is reproduced from (Shivakumara et al., 2017) with permission from the corresponding authors.

SvGs are highly active during the early infection of RKNs. To enter the root, RKNs must degrade the plant cell wall, a complex structure supporting cell growth and development. It acts as a primary physical barrier to plant pathogens and consists of cellulose crosslinked with hemicellulose bound to a pectin matrix (Houston et al., 2016). To invade this complex structure, pre-parasitic RKNs produce various PCWDEs in SvGs which are secreted through the stylet (Figure 3A). Once inside the root, the juveniles start moving through the intercellular space towards the vascular bundle. After locating a suitable site, they become sedentary and begin the formation of GCs. A multitude of effectors is released during this stage from SvGs aiding the initiation and establishment of GCs.

### PCWDEs Mediated Entry of RKNs in the Roots

Cellulase is the first PCWDE identified from the pre-parasitic juveniles (Rosso et al., 1999; Béra-Maillet et al., 2000; Ledger et al., 2006; Hu et al., 2013). It hydrolyses the β-1, 4 glycosidic linkages of cellulose, the major component of the cell wall. Further, pectate lyase and polygalacturonase degrade the pectin matrix (Table 1; Doyle and Lambert, 2002; Jaubert et al., 2002a; Huang et al., 2005a; Chen et al., 2021). Pectate lyase results in an eliminative cleavage of pectin, whereas polygalacturonase hydrolyses the α-1, 4 glycosidic bonds of pectin. Although these enzymes act on pectin, the side chains of pectin – arabinan and arabinogalactan – limit their access to the pectin backbone. A putative arabinanase has been identified in the RKN genomes, however, it is yet uncharacterized (Danchin et al., 2010). Other than cellulose and pectin, hemicellulose is also present in the plant cell wall, especially in monocots which is degraded by RKN xylanase (Mitreva-Dautova et al., 2006). Together with these enzymes, cellulose-binding proteins (CBPs) are also reported in RKNs (Table 1). These proteins do not have enzymatic activity but show strong binding with cellulose and act as anchors for cellulases (Ding et al., 1998). All the PCWDEs are expressed specifically in the pre-parasitic J2s (Figure 3B). A large number of PCWDEs are conserved across multiple RKN species making them indispensable for root penetration (Table 1). Silencing of CWDEs reduced the root-penetration efficiency of RKNs implying their necessity in the early infection (Adam et al., 2008; Shivakumara et al., 2016). Further, pre-parasitic juveniles also assimilate carbon from root surface while they enter the roots (Shivakumara et al., 2017). This early carbon assimilation supports the idea of host-delivered RNAi control strategies that can provide additional benefit of reducing the nematode burden inside the transgenic host expressing RNAi construct.

**Table 1:** Experimentally identified effectors from the sub-ventral glands of various RKNs.

S. No.	Effector	Functionally characterized in	Effector type	Role in parasitism	References
1.	Cellulase	*M. incognita*, *M. javanica*	PCWDEs	Digest cellulose	Rosso et al., 1999; Béra-Maillet et al., 2000; Ledger et al., 2006; Hu et al., 2013
2.	Pectate lyase	*M. javanica*, *M. incognita*, *M. graminicola*		Digest pectin	Doyle and Lambert, 2002; Huang et al., 2005a; Chen et al., 2021
3.	Polygalacturonase	*M. incognita*			Jaubert et al., 2002a
4.	Xylanase	*M. incognita*		Digest hemicellulose	Mitreva-Dautova et al., 2006
5.	Cellulose-binding protein	*M. incognita*		Anchor for cellulase	Ding et al., 1998
6.	Calreticulin	*M. incognita*	Plant defence modulators	PTI suppression	Jaubert et al., 2002b
7.	Mh-265	*M. hapla*			Gleason et al., 2017
8.	MSP-40	*M. incognita*		Cell death suppression	Niu et al., 2016
9.	ISE-5	*M. incognita*			Shi et al., 2018
10.	2G02	*M. javanica*			Song et al., 2021a
11.	GPP	*M. graminicola*			Chen et al., 2017
12.	TTL-5	*M. javanica*		ROS response suppression	Lin et al., 2016
13.	C-type lectin	*M. graminicola, M. incognita*			Zhao et al., 2021

RKNs have an unprecedented diversity of CWDEs and have about 60 genes encompassing 6 different CWDE families. A study on the evolution of PCWDEs has shown that RKNs have acquired these enzymes by horizontal gene transfer from various soil-dwelling and plant pathogenic bacteria (Danchin et al., 2010). In case of polygalacturonase and pectate lyase, the enzyme orthologs are observed in the bacteria *Ralstonia solanacearum* and *Clavibacter michiganensis, respectively*. Both these bacteria are notorious plant pathogens and share the soil niche and host plants with RKNs (Nandi et al., 2018; Xue et al., 2020). In case of cellulase and xylanase, the closest orthologs are present in soil-dwelling bacteria *Cytophaga hutchisonii* and *Clostridium acetobutylicum, respectively*. The phylogenetic evidence underlined with the sympatric nature of these bacteria with RKNs makes them the most likely donors of PCWDEs (Danchin et al., 2010). Furthermore, in RKNs massive amount of gene duplication events have led to multigenic PCWDE families. Although the gross cell wall composition is similar across different plants; structural and molecular heterogeneity is documented (Zhang et al., 2021). These variations might be the reason for the evolution of multiple isoforms of PCWDEs in various RKN species. The effect of cell wall composition on the isoform expression pattern is yet unexplored. RNAseq of the pre-parasitic J2s latched onto root tips of different host plants, before they enter the roots may shed light on the dynamics of multiple PCWDE isoforms (Figure 3C). Other than cell wall composition, in *M. incognita* CWDEs expression is reported to be regulated by other effectors *viz*., *Meloidogyne* secretory protein (MSP)-1, 18, and 20. Silencing of these effectors showed transcriptional oscillation of various CWDEs which suggests the presence of effector-dependent retrograde signalling in RKNs (Shivakumara et al., 2016, 2017; Chaudhary et al., 2019a; Somvanshi et al., 2020). The lack of knowledge about CWDE isoform dynamics is one of the major bottlenecks for not achieving complete control of RKN infection *via* silencing of CWDEs. Hence, the retrograde signalling and effector crosstalk needs to be explored further to control CWDEs expression and limit RKN parasitism. Besides, transcriptional changes in pre-parasitic J2s in response to root exudates implies RKNs’ ability to perceive root signals and modulate gene expression (Teillet et al., 2013). It will be interesting to identify the RKN genes activated by root exudates, as it will enrich our knowledge of early events in RKN parasitism.

### Protection of RKNs and GCs From Plant Immunity

**Figure 4:**
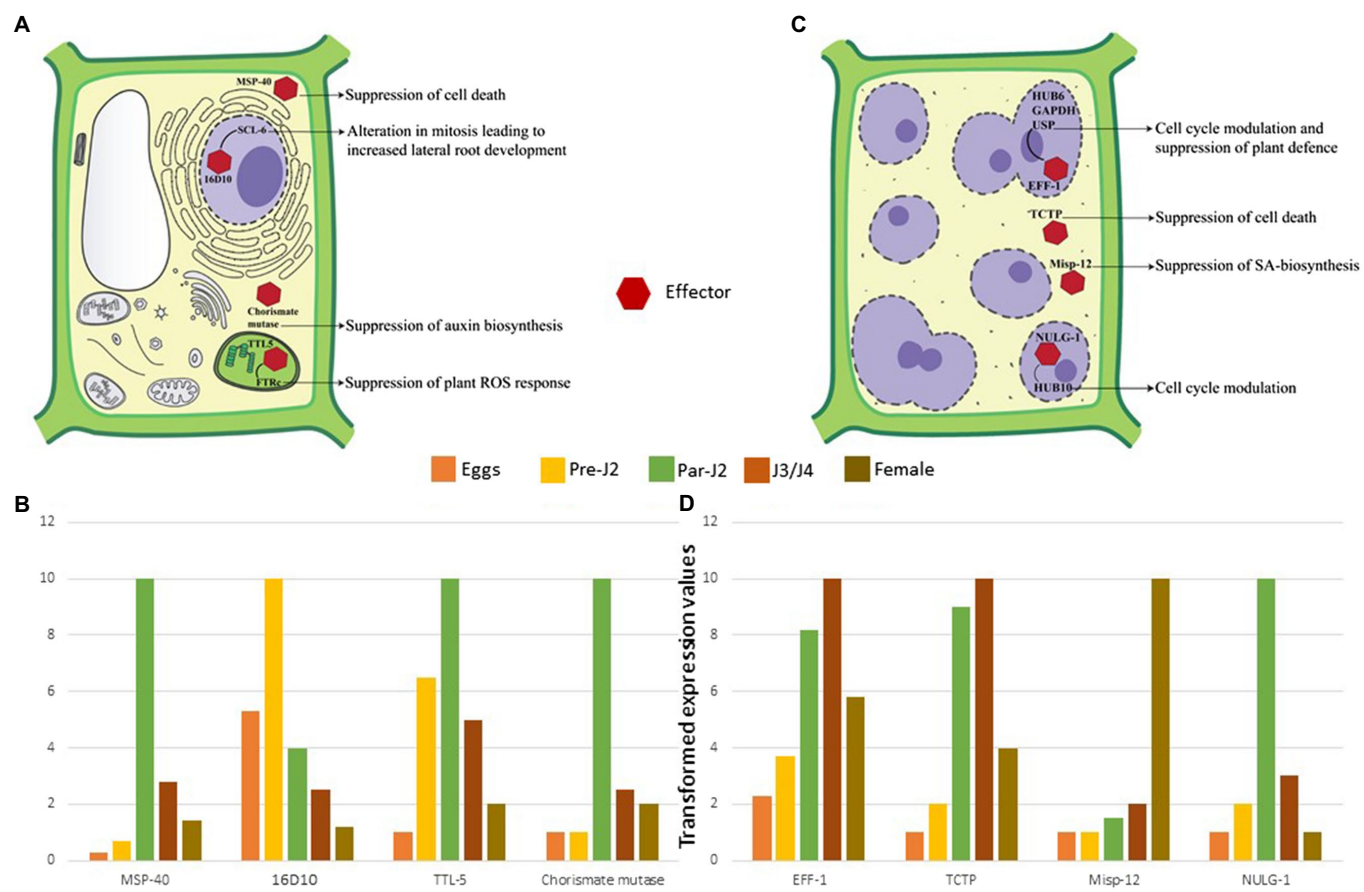
Molecular effects of RKN effectors **(A)** Parasitic juveniles produce effectors in SvGs which modulates plant defence response and cell cycle allowing the formation of GCs. The effectors secreted by parasitic juveniles (shown by red hexagon) target various cellular components and pathways of plants. In the cytoplasm MSP-40 and chorismate mutase suppress the cell death and auxin biosynthesis, respectively. In the plastids, TTL-5 interacts with plant FTRc suppressing the plant ROS response. In the nucleus MSP-16 interacts with plant SCL-6. These interactions result in alteration of mitotic cell division and alternative splicing. **(B)** The expression dynamics reveals that these effectors are produced pre-parasitic and parasitic J2s. Real-time expression data from various articles is used to generate the illustrative patterns of effector gene expression across the different stages of RKN (Huang et al., 2005b, 2006b; Lin et al., 2016; Niu et al., 2016). The maximum expression for a given effector in a particular life-stage is considered as 10. Accordingly, expression at remaining life-stages is calculated which is less than 10. The bars depict these transformed values. Since, these are transformed values from different data sets, it is not possible to give error bars in this case. **(C)** Adult sedentary RKNs produce effectors in DG that help in maintaining GCs throughout the parasitism. Eff-1 interacts with multiple plant proteins in the nucleus leading to cell cycle modulation and RNA instability. Similarly, NULG-1 targeted to the nuclei also help in cell cycle modulation. In the cytoplasm, TCTP and Misp-12 suppress the cell death and salicylic acid biosynthesis, respectively. These effectors and various others help in GC maintenance by suppression of cell death, plant defence response, and cell cycle modulation. **(D)** The expression profile suggests their maximum expression in J3/J4 and adult stages only. Real-time expression data from various articles is used to generate the graph (Xie et al., 2016; Zhuo et al., 2017; Godinho Mendes et al., 2021a,b). The maximum expression for a given effector in a particular life-stage is considered as 10. Accordingly, expression at remaining life-stages is calculated which is less than 10. The bars depict these transformed values. Since, these are transformed values are from different data sets, it is not possible to give error bars in this case.

RKNs are exposed to the host defence system inside the roots which need to be counteracted for survival and establishing a successful interaction with hosts. Furthermore, RKNs being biotrophic parasites, require live plant cells for their feeding. Therefore, they must protect their feeding sites from the host defence system. Pattern triggered immunity (PTI) is the primary defence response of plants against invading pathogens including RKNs, which recognizes pathogen-associated molecular patterns and damage-associated molecular patterns released from the disrupted host tissue. However, the well adapted plant pathogens can counteract PTI by secreting multiple effectors. Hence, PTI is further complemented by effector-triggered immunity (ETI) in which plants recognize the pathogenic effectors that deflect/diffuse the PTI. During RKN attack, plants recognize the ascarosides secreted by pre-parasitic J2s which induces the PTI (Manosalva et al., 2015; Sato et al., 2019). Therefore, RKNs secrete various effectors in the plant apoplast and cytoplasm to interfere with PTI (Table 1). Calreticulin (MiCRT) is the first immune-modulatory effector identified from *M. incognita* which is localized to the plant apoplast where it suppresses PTI (Jaubert et al., 2002b). Although its mode of action is unknown, it is proposed that MiCRT functions as a Ca^2+^ chelating agent. In the plant apoplast, it can prevent Ca^2+^ influx that may suppress the immune signalling. MiCRT silencing reduced the number of galls on plants suggesting its role in successful nematode establishment after root invasion (Jaouannet et al., 2013). Another effector called Mh265 from *M. hapla* is localized to the plant cell cytoplasm and has similar PTI-suppressive effects (Gleason et al., 2017). Other than PTI, hypersensitive response (HR) is another important defence pathway in plants which restricts the spread of pathogens by rapid localized cell death at pathogen penetration site (Morel and Dangl, 1997). It is known that HR hampers GC formation, therefore for survival and successful parasitism, RKNs must suppress HR-mediated cell death (Kumar et al., 2014). Various effectors play a crucial role in cell death suppression. In *M. incognita*, an effector called MSP-40, is localized to the plant cytoplasm and interacts with the mitogen-associated protein kinase pathway leading to the suppression of cell death that protects GCs during development (Figures 4A,B). Moreover, silencing of *MiMsp-40* resulted in a significant reduction in the number of galls indicating its essentiality for parasitism. MSP-40 is observed to be conserved in four other RKN species suggesting it to be an important effector for parasitism (Niu et al., 2016). Another cytoplasm-targeted effector called ISE-5 secreted by parasitic J2s of *M. incognita* suppresses cell death along with basal immune response. Overexpression of *Miise-5* in plants resulted in increased susceptibility to RKN infection indicating its importance in mediating parasitic success. MiISE-5 homologue is observed only in *M. floridiens* suggesting it to be a specialized effector for limited RKNs (Shi et al., 2018). Another effector called 2G02 expressed by pre- and parasitic J2s of *M. javanica* is localized to the plant cell nuclei and is present in all the sequenced RKNs. It suppresses HR-mediated cell death and also reduces the jasmonic acid levels which ultimately enhances the nematode survival (Song et al., 2021b). On the other hand, RKN effector GPP observed only in *M. graminicola* is localized to the plant nuclei where it suppresses cell death helping GCs to survive. The species specificity of GPP suggests the presence of unique infection processes in different RKNs. Silencing of *Mggpp* resulted in fewer adult females signifying its role in nematode development (Chen et al., 2017).

During the early stage of RKN infection, rapid production of reactive oxygen species (ROS) is observed (Melillo et al., 2006). ROS play a crucial role as signalling molecules that activate additional immune responses. RKNs produce at least four effectors – a transthyretin-like protein TTL-5, C-type lectin, protein disulphide isomerase, and Mg16820 – that hinder the ROS response of hosts. TTL-5 studied in *M. javanica* and present in various species of RKNs is produced by parasitic J2s and shows strong interaction with the plant protein FTRc in the plastids. FTRc functions in redox regulation, a part of the antioxidant immune system (Vieira Dos Santos and Rey, 2006). It is seen that MjTTL-5-FTRc interaction increases the ROS-scavenging activity along with the suppression of PTI which increases the susceptibility of plants to RKNs. Silencing of *Mjttl-5* resulted in fewer adult females implying its part in RKN development (Lin et al., 2016; Figures 4A,B). Another effector in this context is C-type lectin that suppresses the ROS response and inhibits PTI, is secreted in the plant apoplast by parasitic J2s. Silencing of C-type lectin decreased the number of penetrating nematodes that suggests its importance in early RKN-plant interactions (Zhuo et al., 2019). Recently, a direct interaction between *M. incognita* C-type lectin and plant catalase was observed. Catalase plays an essential role in H_2_O_2_ homeostasis, and its interaction with C-type lectin points to the fact that the RKNs manipulate the plant ROS response to establish parasitism (Zhao et al., 2021). In addition to these, protein disulphide isomerase, an apoplast-localized effector, plays a crucial role in oxidative stress response. It is reported in *M. incognita* and interacts with stress associated zinc finger protein SAP12, which acts as a redox sensor under oxidizing conditions. Interestingly, SAP12 expression increases significantly during RKN attacks. Therefore, protein disulphide isomerase interaction with SAP12 can potentially regulate redox signalling in plants (Zhao et al., 2020). Additionally, silencing of this effector resulted in the reduction of reproductive ability of nematodes hinting at reduced RKN fitness (Tian et al., 2019, 2020). Another effector called Mg16820 is reported only in *M. graminicola*. It is secreted by parasitic J2s in the plant apoplast and cytoplasm, where it suppresses the ROS synthesis. In the cytoplasm it interacts with dehydration stress inducible protein 1 (DIP-1) that is a crucial abscisic acid responsive gene with functions in biotic and abiotic stress response. The interaction of Mg16820 with DIP-1 may result in suppression of biotic stress response helping RKNs to infect plant roots (Naalden et al., 2018). Other than PTI, cell death, and ROS response modulation, RKN effectors also interact with defence-related proteases in plants (Table 1). In *M. chitwoodi*, Mc1194 an effector expressed by pre-parasitic J2s interacts with plant defence cysteine protease RD21A. However, the results of this interaction on RD21A proteolytic activity are not yet tested experimentally (Davies et al., 2015).

SvG effectors interfere with every major pathway of plant immunity. This allows the formation of GCs during early infection. Currently, the role of very few SvG effectors is known. Future investigations could reveal the functions of other SvG effectors and how they support nematode parasitism. Silencing of these effectors shows various negative impacts on the RKN lifecycle that suggests the loss of fitness of nematodes to overcome active plant immunity. Moreover, the presence of species-specific and multi-species effectors indicates molecular variations in the RKN infection process. Besides the molecular functions of effectors, the RKN immunity network in plants is not well known. Presently, the knowledge about the nematode associated molecular patterns of RKNs, their immune receptors in plants, and the components of RKN-specific ETI is limited. Understanding this molecular network could definitely lay the groundwork for novel RKN-control strategies.

### Hormonal, Transcriptional and Metabolic Changes for GC Ontogenesis

Plant immunity modulation is followed by metabolic and transcriptomic fluctuations. RKNs substantially affect the root morphology suggesting the manipulation of auxin biosynthesis. To achieve this, parasitic J2s secrete an enzyme called chorismate mutase (Lambert et al., 1999; Painter and Lambert, 2003; Huang et al., 2005b; Long et al., 2006). Chorismate mutase is also present in the plants’ plastids and is a part of the shikimate pathway (Romero et al., 1995; Tzin and Galili, 2010). RKNs secrete their chorismate mutase in the plant cytoplasm during early infection which suppresses lateral root development and vascular cell differentiation, a phenotype similar to auxin deficiency (Aloni, 1995; Doyle and Lambert, 2003; Figures 4A, B). It is proposed that RKN chorismate mutase localized to the plant cytoplasm redirects the flux of chorismate from plastids to the cytoplasm. Since chorismate is the precursor of auxin in plastids, the altered flux results in the suppression of auxin biosynthesis arresting the lateral root development (Doyle and Lambert, 2003; Tzin and Galili, 2010; Figure 4A). The widespread presence of chorismate mutase in RKN species suggests that this enzyme is one of the key factors that modulate plant metabolism during infection. Apart from RKNs, plant pathogenic fungi *Ustilago maydis* also secretes chorismate mutase in the host plants during infection. It is observed that fungal chorismate mutase channels the flow of chorismate into phenylpropanoid pathway and prevents the production of salicylic acid (Djamei et al., 2011). Therefore, it will be interesting to study the function of RKN chorismate mutase through metabolic profiling of plants.

Further to hijack the plant cell cycle, RKNs take over plant transcription. A peptide termed 16D10 (also known as MSP-16) is secreted by parasitic J2s (Huang et al., 2003). This short peptide is highly conserved across various RKN species and has a CLAVATA3/Embryo Surrounding Region-Related (CLE) protein domain. CLE peptides are ubiquitous plant peptides involved in stem cell homeostasis (Wang et al., 2016). Interestingly, nematodes are the only animals that produce CLE proteins. In plants, 16D10 interacts with transcription regulators - SCL6 and 21. SCL6 is involved in mitosis regulation, whereas SCL21 has a role in phytochrome signalling and chitin elicitor perception (Day et al., 2003; Yamada et al., 2003; Bolle, 2004; Huang et al., 2006a). The direct interaction of 16D10 with the mitosis regulatory protein has major repercussions on the plant cell cycle and could be the reason for enhanced root development (Huang et al., 2006b; Figures 4A,B). Moreover, silencing of 16D10 showed a significant reduction in the number of galls and eggs across various RKN species. This suggests its fundamental role in parasitism and nematode development making it an attractive target for RKN control (Huang et al., 2006a; Yang et al., 2013; Dinh et al., 2014, 2015). Along with transcription regulators, RKNs impact the alternative splicing of plant transcripts with the help of an effector called EFF-18 (Mejias et al., 2021). In the plant nucleolus, it interacts with a ribonucleoprotein SmD1, a key component of spliceosomal machinery (Mejias et al., 2021). EFF18-SmD1 interaction directly alters the genes involved in auxin and abscisic acid signalling, RNA-binding proteins, and DNA-replication related proteins (Mejias et al., 2021). This suggests the pivotal role of EFF-18 in the reprogramming of plant cells for giant cell ontogenesis.

Apart from affecting immune, metabolic, and transcriptomic pathways, RKNs also remodel the cytoskeletal architecture of plant cells (de Engler et al., 2004, 2010). Parasitic J2s secrete profilin (Leelarasamee et al., 2018) which is a small protein that binds to actin monomers to regulate its homeostasis (Smant et al., 1998; Pernier et al., 2016). RKN profilin affects the polymerization of soluble actin (Leelarasamee et al., 2018). This corroborates well with the presence of fragmented actin filaments in GCs (de Engler et al., 2004; de Engler and Favery, 2011; Liu et al., 2016a). This indicates that RKNs reduce the actin network density in GCs to facilitate their feeding rate. Recently, a nucleus-targeted effector called Minc00344 was seen to interact directly with HUB-10, a kinesin light chain-related protein. It is known that HUB-10 is involved in maintaining the stability of cortical microtubules in *Arabidopsis* which ultimately help in maintaining the plant cell growth and shape (Liu et al., 2016b; Ganguly et al., 2020). Therefore, Minc00344-HUB-10 interaction may result in GC growth and shape modulation (Godinho Mendes et al., 2021a). Since GCs are multinucleated and act like metabolic sink for RKNs, they require a high supply of water and other solutes for sustenance to support the feeding nematodes (Rodiuc et al., 2014). Parasitic J2s secrete 8D05 (also known as MSP-9) that interacts with a plant aquaporin called tonoplast intrinsic protein 2 (TIP-2) – a tonoplast-located water channel (Xue et al., 2013). TIP-2 is involved in water transport by enhancing water permeability and is essential for the transport of urea and extracytosolic ammonia across the tonoplast membrane (Daniels et al., 1996; Liu et al., 2003; Leitão et al., 2012; Xue et al., 2013). As ammonia plays a principal role in plant metabolism, the regulation of its transporters is equally important for plant cell development (Loqué et al., 2005). As GCs show high metabolic activity, increased solute concentration can result in high turgor pressure, thus requiring proper regulation to maintain cell integrity. Therefore, the interaction of MSP-9 with TIP-2 suggests its role in regulating the transport of water and other nutrients across GCs (Xue et al., 2013).

Though several other SvG effectors are known, their role in infection is yet to be deciphered. A protein belonging to SXP/RAL-2 superfamily is identified in parasitic J2s of *M. incognita* (Tytgat et al., 2005). SXP-RAL-2 proteins described as being secreted by animal-parasitic nematodes into their hosts interact with the host immune system, however, its role in RKNs in yet unknown (Kobayashi et al., 2007). An aspartic-like protease is secreted by parasitic J2s in the plant apoplast. It is hypothesized to degrade the plant proteins involved in defence (Vieira et al., 2011). MSP-3 which shows high expression in parasitic juveniles has a putative pentein domain that may take part in the translational regulation and cell signalling. Its silencing resulted in reduced number of galls and mature females signifying its involvement in RKN development (Joshi et al., 2020). Another protein known as MSP-2 produced by parasitic J2s of *M. incognita* contains the ShK toxin domain. Proteins with this domain are known to block the K^+^ channels. Therefore, MSP-2 is proposed to be involved in blocking plant K^+^ channels. Silencing of *Mimsp-2* causes substantial developmental retardation in RKN females implying its need in nematode development (Joshi et al., 2019). Venom allergen-like proteins (VAPs) are also synthesized by pre-and parasitic J2s (Ding et al., 2000; Wang et al., 2007). VAPs from animal parasitic nematodes are involved in host immunomodulation (Bower et al., 2008). Furthermore, VAP from cyst nematode *Globodera rostochiensis* target the apoplastic cysteine protease Rcr3^pim^ and results in the loss of basal immune response of host plants (Lozano-Torres et al., 2014). However, their functions in RKNs are still enigmatic. The silencing of VAP resulted in the decline of RKN development and reproduction (Chaudhary et al., 2019b). It further hampered the early-stage infection behaviour by altering the PCWDEs expression (Duarte et al., 2017; Chaudhary et al., 2019a). This suggests its role as a regulator of PCWDEs. Expression of 2 VAPs was observed in *M. graminicola* trying to infect RKN-resistant rice plants, suggesting their function to counteract host immunity (Petitot et al., 2020). Similarly, silencing of *msp-20* in *M. incognita* had negative effects on PCWDEs expression. It also hampered nematode development, behaviour, and cellular physiology by disturbing various developmental signalling pathways (Shivakumara et al., 2017; Somvanshi et al., 2020). This clearly suggests the pleiotropic effects of effector silencing on RKN fitness (Somvanshi et al., 2020). Therefore, it will be interesting to study *in vivo* functions of these effectors and targeting effectors with crucial molecular functions for RKN control.

RKN SvGs are active during the early stage of infection. To date, many effectors are studied at the pre-parasitic stage. However, the effectors released by parasitic J2s are still under investigation. A detailed experimental analysis of all these effectors is necessary to elucidate the molecular remodelling occurring in plant cells during the process of parasitism. Further, it is necessary to understand the transcriptional regulation of these effectors in RKNs. Detailed pan-omics analysis along with functional studies will provide a better insight into sptio-temporal regulation and molecular mechanism of these effectors. Identification of master cis- or trans-regulatory elements of SvG effectors will help in controlling the RKN infection at early stages.

## Maintenance of Gcs in Late Infection Requires Secretions From Dg

After becoming sedentary and initiating feeding site formation, RKNs undergo moulting through non-feeding J3, J4 and the adult female stage that start feeding on the GCs. During this period slowly SvGs functioning reduces and DG becomes active. DG effectors are involved in two major functions: plant cell cycle modulation and suppression of cell death (Table 2). EFF-1, a nuclear localized effector is expressed in J3/J4 and females of *M. incognita* and present in multiple RKN species (Jaouannet et al., 2012). In the GC nucleus, it interacts with three plant proteins namely HUB-6, glyceraldehyde 3-phosphate dehydrogenase (GAPDH), and universal stress protein (USP; Godinho Mendes et al., 2021b; Truong et al., 2021; Figure 4B). HUB-6 is an important transcription factor that regulates the cell cycle, plant development, and plant immune response (Li, 2015). This suggests that HUB-6 is a major target for parasitism and the modifications upon binding EFF-1 abets nematodes to suppress plant defence, regulate the cell cycle, and maintain GCs (Godinho Mendes et al., 2021b). GAPDH is one of the most important housekeeping enzymes. It is a moonlighting protein involved in glycolysis, apoptosis, autophagy, gene expression regulation, and response to stress (Tristan et al., 2011). It is known that cytoplasmic GAPDH re-localizes to the nucleus under oxidative stress and protects nucleic acids. The ROS response generated during RKN infection could target GAPDH to GC nuclei and the interaction of EFF-1 with GAPDH suggests an alteration in its nuclear function of nucleic acid protection (Truong et al., 2021). USP is a multifunctional protein involved in biotic and abiotic stress responses and in the nucleus, it binds to RNA to protect it during environmental stress (Chi et al., 2019). EFF-1-USP interaction in the nucleus suggests that it affects the RNA-protective function of USP which may facilitate transcriptional modulation in GCs (Truong et al., 2021). Further transcriptomic study identified another effector localized to the GC nuclei, namely a small glycine cysteine-rich-1 (Table 2). This protein has no known functional domains and is highly expressed during J3 and J4 stages (Figure 4B). In plants, it inhibits cell death suggesting its role in maintaining GCs during non-feeding stages of parasitism. Additionally, its silencing showed a substantial decrease in the number of eggs indicating the reduced fitness of RKNs due to defects in GC functioning (Nguyen et al., 2018). NULG-1 expressed only in parasitic stages is a highly conserved effector across RKN species and its overexpression in plants increased their susceptibility to RKNs (Table 2). Further, its silencing resulted in fewer galls, eggs, and parasitic nematodes in roots thus underpinning its critical role in parasitism (Wu et al., 2012). Recently it was seen that NULG-1 interacts with a plant HUB-10 that is involved in the regulation of the plant defence response (Bürstenbinder et al., 2013). The *Hub-10* mutant plants showed substantial increase in the number of RKN galls and eggs, clearly suggesting its involvement in defence against RKNs. The interaction of NULG-1 with HUB-10 may neutralize its defensive actions aiding the RKN infection (Godinho Mendes et al., 2021a; Figures 4C,D). An effector from *M. graminicola* called as Mo237 is secreted into plant nucleus and cytoplasm by J3 and J4 stages. This effector is observed in multiple RKN species and it interacts with 3 plant proteins – 1,3-β-glucan synthase component, cysteine-rich repeat secretory protein 55, and pathogenesis-related Betvl family protein – all of which are involved in plant defence. Interaction of Mo237 with these plant proteins results in suppression of plant-defence genes, cell wall callose deposition, and ROS burst signifying its role in the suppression of basal plant immunity during later parasitic stages. Mo237 overexpression in plants resulted in about 67% increase in the adult female number indicating its necessity in parasitism and RKN development (Chen et al., 2018). Another effector Mo289 expressed by J3 and J4 of various RKNs interacts specifically with a copper metallochaperone heavy metal-associated plant protein 04 in the host cytoplasm and nucleus. This interaction disrupts the activity of Cu/Zn superoxide dismutase resulting in overall suppression of ROS response. Further, it also results in suppression of cell death suggesting its role in maintenance of GCs. Overexpression of Mo289 in plants showed increased susceptibility to RKN infection, whereas RNAi of the same effector led to decrease in the number of females implying its importance in nematode development and parasitism (Song et al., 2021a).

**Table 2:** Experimentally identified effectors from the dorsal gland of various RKNs.

S. No.	Effector	Functionally characterized in	Effector type	Role in parasitism	References
1.	EFF-1	*M. incognita*	Cell cycle modulator	Plant cell cycle modulation	Godinho Mendes et al., 2021b
2.	NULG-1	*M. javanica*	Plant defence modulator	Plant defence suppression	Godinho Mendes et al., 2021a
3.	Mo237	*M. graminicola*			Chen et al., 2018
4.	Mo289	*M. graminicola*		ROS response suppression	Song et al., 2021a
5.	MSP-18	*M. incognita*		Cell death suppression	Grossi-de-Sa et al., 2019
6.	SGCR-1	*M. incognita*			Nguyen et al., 2018
7.	TCTP	*M. enterolobi*			Zhuo et al., 2017
8.	MiSP-12	*M. incognita*	Plant hormone regulator	SA-biosynthesis suppression	Xie et al., 2016

Although some effectors target nuclear processes, others remain specifically in the cytoplasm and aid in GC maintenance. Translationally controlled tumour protein (TCTP) is a highly conserved multifunctional eukaryotic protein involved in spindle formation, modulation of cell growth signalling pathways, and anti-apoptosis (Bommer, 2017). It is found in the secretions of animal-parasitic nematodes and helps them in reproduction, adaptation to stress, and modulation of the allergic inflammatory response (Gnanasekar et al., 2002; Mak et al., 2007; Meyvis et al., 2009). In *M. enterolobii*, TCTP is highly expressed in J3s and secreted in the plant cytoplasm where it suppresses cell death (Figures 4C,D; Bellafiore et al., 2008; Zhuo et al., 2017). Thus, MeTCTP is an important effector for GC maintenance. Another cytoplasmic effector named Misp-12 is expressed specifically in females. Its silencing resulted in a sharp decrease in J3/J4 and female population in plants implying its role in nematode maturation. Interestingly, it suppresses salicylic acid biosynthesis as it harms the nematode lifecycle (Xie et al., 2016; Figures 4C,D).

Other than effectors described above, functions of a large number of DG effectors are yet to be explored (Table 2). Overexpression of MSP-7 in plants accelerated the formation of GCs and increased the number of RKN eggs. It is speculated to have an important role in establishing a compatible interaction between hosts and nematodes (de Souza et al., 2011). An apoplast-targeted effector 6D4 is secreted by the sedentary stages of RKNs. Its exact involvement in parasitism is unknown, however, it may have a role in giant cell maintenance (Vieira et al., 2011). MSP-18 is expressed in all the parasitic stages of nematodes with maximum expression in J3/J4. The silencing of MSP-18 resulted in a significant decrease in the nematode population. Further, it also reduced the expression of pectate lyase and polygalacturonase suggesting its crosstalk with CWDEs (Shivakumara et al., 2017). Besides, MSP-18 overexpressing plants are more susceptible to nematode infection and it also suppresses the plant cell death that may help in GC maintenance (Grossi-de-Sa et al., 2019).

DG is highly active in the later stages of RKN parasitism. However, the molecular switch that suppresses SvGs and activates DG is yet to be discovered. Recently, a DNA motif named Mel-DOG was identified upstream of many DG effectors suggesting it to be their cis-regulatory motif. Although its functional characterization is still ongoing, it will be interesting to target such genomic motifs to suppress multiple genes using novel genome editing technologies that are being developed for parasitic nematodes. (Da Rocha et al., 2021; Eves-van den Akker et al., 2021; Kranse et al., 2021). Possibility of targeting functionally similar and diverse genes for silencing in *M. incognita* using a fusion gene construct has been demonstrated recently (Banakar et al., 2020 Hada et al., 2020 2021).

## Non-Oesophageal Effectors: a New Layer of Complexity To Endoparasitism

RKNs secrete some effectors from body parts other than oesophageal glands (Table 3). Amphids are the first non-oesophageal organ reported to secrete effectors (Semblat et al., 2001). Amphids are the anterior sensilla of the RKNs. Amphidal secretions are thought to be involved in chemoreception (Perry, 1996). A protein called Meloidogyne avirulent protein (MAP)-1 is a putative secretory protein from amphids (Semblat et al., 2001; Castagnone-Sereno et al., 2009) and is localized to the plant apoplast during early parasitism. It is hypothesized to be involved in the induction of GCs (Vieira et al., 2011). Further bioinformatic analyses showed the presence of an endoglucanase domain in MAP-1 implying its involvement in cell wall digestion (Adam et al., 2009). Furthermore, various repetitive regions are identified in MAP-1 sequences which suggest its involvement in direct interaction with plant ligands and GCs wall. However, all these speculations remain to be proved experimentally (Semblat et al., 2001; Castagnone-Sereno et al., 2009). Another amphidal effector observed in *M. hapla* is MhTTL2 containing a transthyretin-like protein domain. However, its exact function in parasitism is not understood yet (Gleason et al., 2017). Another interesting effector, Minc00801 is observed in the rectal glands of adult females (Rutter et al., 2014). Since rectal glands function in producing the egg mass matrix, this effector may have some role in nematode reproduction. Corroboratively, silencing of Minc00801 resulted in a substantial decrease in the number of galls suggesting its importance in the overall RKN lifecycle (Danchin et al., 2013). In *M. javanica*, a fatty acid and retinol binding protein (FAR-1) is secreted by sedentary stages into host plants through the cuticle. Overexpression of *Mjfar-1* in tomato resulted in enhanced susceptibility of plants to RKNs and produced larger GCs compared to control. Moreover, its silencing showed reduction in RKN development. It was also observed that *Mjfar-1* overexpression resulted in substantial downregulation of JA-responsive genes, implying its role in suppression of immune response (Iberkleid et al., 2013). Recently, a macrophage migration inhibitory factor (MIF) was identified to be secreted from the hypodermis of RKNs (Zhao et al., 2019). MIFs are well studied in mammals and are known to be involved in inflammation and innate immune responses (Xu et al., 2013). In animal-parasitic nematodes, MIF-like proteins are involved in the immune escape from host cells. MIF of hookworms interacts with CD74 of human cells which facilitates larval infection and development (Cho et al., 2007). In parasitic J2s their expression is maximum, and they are secreted in the giant cells. *In planta*, MIF interacts with annexin, a central regulator of plant growth and stress signalling. It also suppresses PTI as well as PCD which can aid in nematode infection (Zhao et al., 2019).

**Table 3:** Experimentally identified non-oesophageal effectors from various RKNs.

S. No.	Effector	Secreted from	Observed in	Putative functions	References
1.	MAP-1	Amphids	*M. incognita*	Induction of GCs, Digestion of cell wall	Semblat et al., 2001; Castagnone-Sereno et al., 2009; Vieira et al., 2011
2.	TTL-2		*M. hapla*	–	Gleason et al., 2017
3.	Minc00801	Rectal gland	*M. incognita*	Involved in RKN reproduction	Rutter et al., 2014
4.	FAR-1	Cuticle	*M. javanica*	JA-suppression	Iberkleid et al., 2013
5.	MIF	Hypodermis	*M. incognita*	Suppression of PTI and cell death	Zhao et al., 2019

Non-oesophageal effectors of RKNs are still an uncharted area of research. With new transcriptomic datasets and microscopy techniques, these effectors are being discovered. The molecular complexity evolved by RKNs to hijack plant cells is intriguing. Such a high degree of evolution makes these microscopic worms one of the most successful plant endoparasites and very challenging to understand.

## Species-Specific and Host-Specific Effector Dynamics

The genus *Meloidogyne* is very diverse and divided into 3 groups based on the mode of reproduction (Karssen and Moens, 2006). However, the overall infection process of all the species is similar. Various effectors of *M. incognita* are highly conserved across different species suggesting a common infection mechanism (Da Rocha et al., 2021). Nevertheless, some species show the presence of unique effectors. The 48 non-redundant SvG effectors and 34 DG effectors of *M. incognita* are conserved in *M. arenaria, M. javanica*, and *M. enterolobii*, all of which are polyploid and parthenogenetic species. On the other hand, only 25 SvG effectors and 8 DG effectors are conserved in *M. hapla* which is diploid and facultative parthenogenetic RKN. This suggests a probable link between the mode of reproduction and effector diversity in RKNs (Da Rocha et al., 2021). Currently, the genomic data is available for only 7 species which makes it difficult to identify any host-specific or species-specific effectors. Still a limited number of such effectors are observed. GPP, an SvG effector, is observed only in *M. graminicola* which infects plants of *Poaceae* family, suggesting a plant-specific infection pathway (Chen et al., 2017). Similarly, *M. chitwoodi* has a cysteine protease inhibitor that helps in inhibition of host defence proteases (Davies et al., 2015). *M. chitwoodi* mainly infects plants of *Solanaceae* family, therefore the presence of a unique effector suggests a host-specific infection mechanism. Similarly, DG-encoded MSP-6 is also unique to *M. incognita*, the function of which is yet undetermined (Da Rocha et al., 2021).

In case of resistant and susceptible RKN plants, changes in the effector dynamics are observed. In RKN resistant rice plants, effector genes of *M. graminicola* were expressed for a relatively long time as compared to susceptible plants. Moreover, two VAPs were specifically induced during infection of resistant plants reflecting their necessity to counteract host defence system (Petitot et al., 2020). Similar results were also observed in case of *M. incognita* feeding on susceptible and resistant tomato plants. In case of resistant plants substantial upregulation of various effectors was observed suggesting their involvement in establishing infection (Shukla et al., 2018). This suggests that RKNs can tweak their effector expression according to the host plant. It will be interesting to study the effector dynamics of RKN species in different host and non-hosts as well as resistant and susceptible plant lines to further understand the effector diversity and dynamics.

## Conclusion and Future Prospects

RKNs are unique plant-parasitic nematodes that cause severe losses in plant productivity. It is known that for successful parasitism, RKNs secrete a repertoire of effectors that manipulate host physiology, development and immunity. These effectors are expressed in a timely manner during infection. As pre-parasitic J2s invade the cell wall barrier, a myriad of CWDEs is upregulated during eggs to pre-parasitic J2 transition. Once inside the root, RKNs become sedentary, undergo moulting, and initiate GC progression. These developmental changes are demarcated by a sharp increase in the expression of secretory enzymes and proteins involved in stress tolerance and plant immunity modulation (Da Rocha et al., 2021). Most of the studies till date have focussed on the effects of a single effector at a time. However, it is evident that RKNs deploy multiple effectors to achieve parasitism and we need to look at the orchestral effect of all the effectors in order to elucidate their infection process. Recently, for the first-time multiple effector silencing was achieved in *M. incognita* using a fusion gene construct to silence functionally diverse genes (Hada et al., 2021). Simultaneous knockdown of three effectors namely, MSP-1, 18, and 20 resulted in the reduced nematode burden in plants. Furthermore, the fusion gene construct showed better performance than single gene knockdown. Thus, this strategy opens the door for simultaneous knockdown of multiple functional genes of RKNs to develop highly resistant plants (Hada et al., 2021). Moreover, a few months back in a ground-breaking report, delivery and expression of exogenous nucleic acids in juvenile and adult plant-parasitic nematodes was shown (Kranse et al., 2021). This research builds the foundation for genetic manipulation of plant-parasitic nematodes and opens up a huge avenue to study the molecular biology of the sedentary endoparasitic nematodes that threaten the global agriculture.

From the available reports, we have obtained several exciting molecular dynamics that happen during the parasitism through effector molecules not only in the plants but also in the nematodes. Interestingly, the expression profile of candidate effectors in the nematode life cycle sheds light on their specific function(s). For example, the tactics by which RKNs hijacks the plant cell cycle, metabolism, and suppress defence to promote growth and reproduction is exciting and systematically programmed. In this respect, the functions of effectors secreted by SvGs and DG are well coordinated. It will be interesting to know whether such molecules are present in other plant and animal nematodes. Molecular insights obtained from species-specific and host-specific effectors will reveal unique parasitism pathways. Furthermore, early-stage carbon assimilation by pre-parasitic juveniles makes them susceptible to root surface mediated RNAi control strategies. We suggest the following studies will be very much relevant and necessary on RKNs and plants concerning their compatible and non-compatible interactions

1. Role of RKN effectors in host and non-host plants to unravel specificity.
2. Profiling of effector expression during the infection process in diverse hosts prior to root entry to understand the importance and role of PCWDE isoforms.
3. Deciphering the rationale for the presence of such large number of effectors across RKN species.
4. Characterization of promoters, cis/trans regulators and other transcriptional regulators of RKN effectors and their secretion/activation.
5. Nematode associated molecular patterns and discovery of elicitors and their perception by plants to activate specific defence in nematode-resistant lines.
6. Development and utilization of tools for functional characterization of nematode molecules.
7. Retrograde signalling in nematode.
8. Understanding and employing simultaneous knockdown of multiple effector genes of RKNs to increase plant protection.
9. Utilization of RKN-inducible root specific promoters to generate novel root mediated RNAi plants.
10. RKN genome sequencing for identification of species-specific effectors.

Since effectors are the implacable molecular weapons that RKNs harbour to invade the plant, it is extremely important to understand their functions to design counter-strategies against their infection to control losses and increase the productivity in agricultural and horticultural crops.

## Author Contributions

AG and UR conceptualized the idea. SJ wrote the draft. All authors read and edited the manuscript.

## Funding

Financial support received from Department of Biotechnology, Government of India through grant number BT/PR23640/AGIII/103/1043/2018.

## Conflict of Interest

The authors declare that the research was conducted in the absence of any commercial or financial relationships that could be construed as a potential conflict of interest.

## Publisher’s Note

All claims expressed in this article are solely those of the authors and do not necessarily represent those of their affiliated organizations, or those of the publisher, the editors and the reviewers. Any product that may be evaluated in this article, or claim that may be made by its manufacturer, is not guaranteed or endorsed by the publisher.
